# Analytical Quality by Design: Achieving Robustness of an LC-CAD Method for the Analysis of Non-Volatile Fatty Acids

**DOI:** 10.3390/ph16040478

**Published:** 2023-03-23

**Authors:** Rasmus Walther, Jovana Krmar, Adrian Leistner, Bojana Svrkota, Biljana Otašević, Andjelija Malenović, Ulrike Holzgrabe, Ana Protić

**Affiliations:** 1 Institute for Pharmacy and Food Chemistry, University of Würzburg, Am Hubland, 97074 Würzburg, Germany; 2 Department of Drug Analysis, Faculty of Pharmacy, University of Belgrade, Vojvode Stepe 450, 11 221 Belgrade, Serbia

**Keywords:** Analytical Quality by Design, fatty acids, charged aerosol detector, polysorbate 80, magnesium stearate

## Abstract

An alternative to the time-consuming and error-prone pharmacopoeial gas chromatography method for the analysis of fatty acids (FAs) is urgently needed. The objective was therefore to propose a robust liquid chromatography method with charged aerosol detection for the analysis of polysorbate 80 (PS80) and magnesium stearate. FAs with different numbers of carbon atoms in the chain necessitated the use of a gradient method with a Hypersil Gold C_18_ column and acetonitrile as organic modifier. The risk-based Analytical Quality by Design approach was applied to define the Method Operable Design Region (MODR). Formic acid concentration, initial and final percentages of acetonitrile, gradient elution time, column temperature, and mobile phase flow rate were identified as critical method parameters (CMPs). The initial and final percentages of acetonitrile were fixed while the remaining CMPs were fine-tuned using response surface methodology. Critical method attributes included the baseline separation of adjacent peaks (α-linolenic and myristic acid, and oleic and petroselinic acid) and the retention factor of the last compound eluted, stearic acid. The MODR was calculated by Monte Carlo simulations with a probability equal or greater than 90%. Finally, the column temperature was set at 33 °C, the flow rate was 0.575 mL/min, and acetonitrile linearly increased from 70 to 80% (*v*/*v*) within 14.2 min.

## 1. Introduction

For decades, pharmaceutical analysis was essentially a discipline in which indispensable chromatographic methods were developed on a trial-and-error basis. In recent years, however, advances in computational tools have increasingly enabled analysts to efficiently select the most appropriate separation conditions, taking into account the entire experimental space. Due to its resource-conscious nature, chemical intelligence has been gaining momentum and is now a key dimension of both the established Pharma 4.0 as well as emerging Pharma 5.0 industry perspectives.

As the heart of the pharmaceutical industry, new digital technologies are bringing prosperity, improved process understanding, and better time management across all segments, with a special focus on already overburdened quality control labs [1,2,3,4]. A particular benefit is the supply of the more consistent quality method and ultimately patient safety, as failure of the analytical method can have dramatic implications for human health [5]. Consequently, a variety of in silico solutions for selecting fit-for-purpose analytical methods have been developed to date.

Analytical Quality by Design (AQbD) is one of the powerful assays in the arsenal of computational tactics that labs with extensive responsibilities and finite resources have been using to develop analytical methods [6,7,8,9,10,11,12,13]. The final result of AQbD is a Method Operable Design Region (MODR), which is a multidimensional space based on the experimental settings that ensure suitable method performance. Changes within the MODR do not need to be submitted to regulatory agencies, which saves time and reduces the potential for analytical method failure in quality control labs [3]. AQbD has been included in the guidelines of the International Conference on Harmonization of Technical Requirements for Pharmaceuticals for Human Use, specifically the ICH Q2 (R2) (analytical validation) and the new ICH Q14 (analytical procedure development) [1,3,14].

Unfortunately, the recognizable systematic approach outlined here is in some respects contrary to the approach used to analyze many excipients in the *European Pharmacopoeia* (Ph. Eur.). This is particularly problematic when it comes to a large group of excipients counting polysorbates (PS), esters of macrogol or glycerol with various fatty acids (FA), or mineral salts of FAs [15,16,17,18]. Typically, the FA composition of these excipients is determined through gas chromatography (GC) after a laborious process of derivatization to the respective volatile methyl esters. According to the monograph 2.4.22 “Composition of fatty acids by gas chromatography” in the Ph. Eur. [19], FAs with chain lengths of 6–24 carbon atoms can be analyzed only if they do not contain thermolabile moieties, such as an epoxy group [20]. The fact that GC analysis endures for about 60 min, requires a larger amount of expensive carrier gases such as helium or hydrogen, and consumes valuable time highlights the need for improvement in this area.

In this regard, Ilko et al. [21] developed a liquid chromatography (LC) method with the charged aerosol detector (CAD) as an alternative to the compendial GC method for the analysis of PS 80. The CAD’s capability to detect analytes irrespective of their chemical structure enabled the convenient analysis of FAs without derivatization, with a shorter runtime of just 18 min. However, the analytical method encountered sensitivity issues with short-chain FAs and was not developed through a systematic risk-based approach. Recent research has explored the suitability of a new generation of CAD instruments for the sensitive detection of non-volatile FAs [22]. This study has taken into account adjustable evaporation temperature, filter constant, and power function value (PFV). The existing knowledge was used in this work to improve the limitations of the previously developed HPLC methods with CAD [21,22] and combine them with the advantages of the AQbD approach.

**Figure 1:**
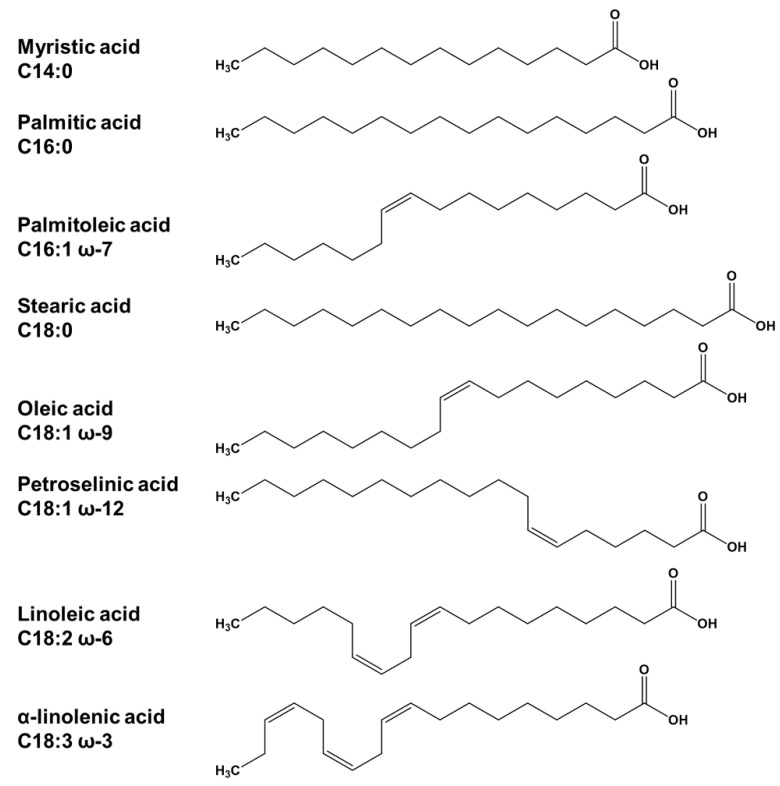
Common names, lipid numbers, and molecular structures of the analyzed non-volatile fatty acids.

Applying AQbD not only provides an in-depth comprehension of the analytical process, but also ensures the robustness and long life cycle of the methods. These are all attributes that are particularly desirable for general compendial methods such as FA composition. Our aim was therefore to use the AQbD approach to identify the critical factors of the existing methods [21,22] and to optimize them in such a way that a sensitive and, above all, accountable LC-CAD method with appropriate risk management is available. To demonstrate the adequacy of the new method with the Ph. Eur. GC method for the determination of FA composition, a magnesium stearate batch was investigated in addition to a Polysorbate (PS) 80 sample as a further application example (Figure 1).

## 2. Results and Discussion

### 2.1. Analytical Target Profile: Defining the Scope of the Method

AQbD workflow begins by summarizing the characteristics of the analytical method that ideally will be achieved to guarantee the desired quality. The first step of this dynamic procedure thus includes defining the analytical target profile (ATP). The concept of an ATP is in line with the concept of the quality target product profile defined in the ICH Q8 guideline and was recently introduced through the ICH Q14 guideline [3,23]. The ATP involves a description of the method purpose, the selection of an appropriate analytical technique that is able to serve this purpose, and finally, the selection of the quality attributes that are going to be measured along with method performance characteristics. Therefore, the declaration of the ATP represents the base step enabling further definition of desirable analytical method attributes with associated acceptance criteria [3]. In the specific case presented in this study, achieving the baseline separation of eight non-volatile FAs (see Figure 1) in a sufficiently short analysis run-time posed the purpose of the intended LC-CAD method.

### 2.2. Design of Experiments in Modeling of Critical Method Attributes

**Figure 2:**
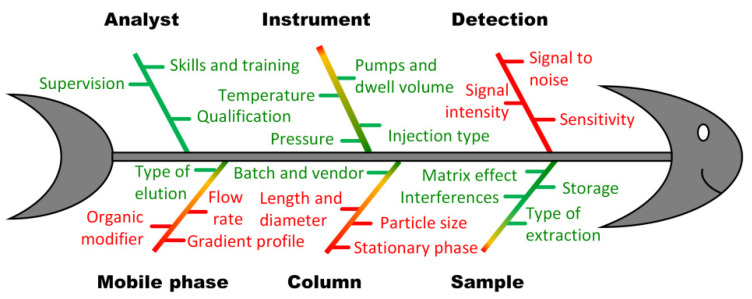
Fish bone diagram with parameters affecting the quality of LC analyses.

The essence of the ATP was more precisely presented by defining critical method quality attributes, called critical method attributes (CMA), associated with their acceptance criteria. The selection of CMAs refers to a set of chromatographic separation descriptors that are going to be used as a proper indicator of the capability of a method to reach the predefined ATP. In LC, these method attributes are derived from the separation of critical pair of analytes, specific requirements for the peak shape, and/or the number of theoretical plates, etc., and may vary depending on the method purpose [24]. Accordingly, CMAs are influenced by the wide range of LC system operating parameters, which are therefore accordingly denoted as critical method parameters (CMP) [3,6,7,10]. In general, LC method parameters may be classified in a way that all aspects of intended LC analysis, such as sample, mobile phase, detection, or column-related parameters, are properly taken under consideration (Figure 2). The analysis of plotted Ishikawa or fish bone diagram followed with an appropriate risk-based approach or based on previous knowledge was used to provide comprehensive method understanding, as well as a scientifically based definition of CMPs. Using an approach known as CNX, important decisions were made about which method parameters should be kept under **C**ontrol, which could be considered **N**oise factors, and finally, which method parameters required e**X**perimental evaluation of the associated acceptable ranges. The parameters that needed to be kept under control were set to constant values. Apart from providing proper insight into the intended method properties, this approach provides inputs for prospective method control strategy [25,26].

Design of Experiments (DoE), as one of the constitutive concepts of the AQbD paradigm, represents an excellent in silico tool for the resource-efficient development of various chromatographic methods. Primarily, DoE provides high-quality information as a consequence to the simultaneous variation of experimental LC-CAD variables. However, when the number of factors to be considered raises, the increase of the cost and the time needed for the analyses follows as well. This is especially evident when categorical variables (e.g., type of column) are involved. Adjusting all the potentially influential factors, thus, almost certainly ends up with the significant quantity of runs that is impractical to be carried out [27]. Alternatively, since profound background knowledge is available, the parameter optimization can be accomplished through a multistage strategy that includes the following: (1) preliminary scouting of the categorical factors, namely, column chemistry and solvent type; (2) fixing some of the high-risk factors at levels that ensure the fulfillment of practical requirements; (3) fine-tuning of secondary high-risk factors via Response Surface Methodology (RSM) design; (4) construction of the MODR for robust method performance [28]. This sequential strategy provides also independent assessment of potential interactions between the significant factors for a better understanding of the method.

#### 2.2.1. Scouting Stage: Selection of Organic Solvent and Column Type

It is known that the chemistry of the stationary phase and the type of organic modifier are primary factors in determining the retention behavior of compounds in reversed phase (RP) chromatography [24]. In this study, the type of organic modifier (ACN) was selected a priori. This decision was supported by the fact that ACN-based mobile phases generally have high elution strength, which is beneficial when working with lipophilic analytes such as FAs. Nevertheless, a better evaporation profile over frequently used MeOH was meaningful since volatility is a very desirable characteristic in terms of the adopted detection technique [29].

**Figure 3:**
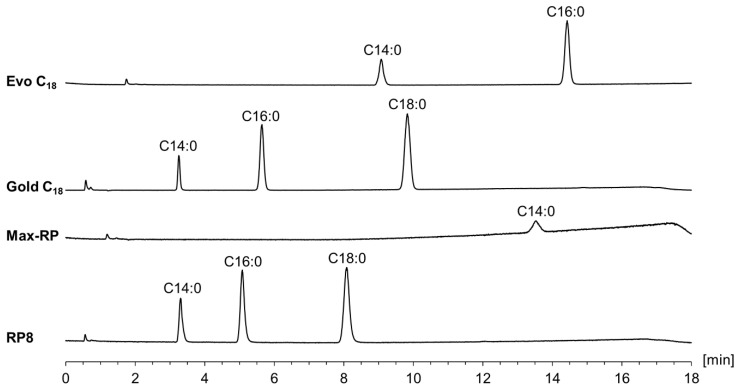
Chromatogram overlay of the column screening using the gradient program of Ilko et al. [21] (see Table S1). Injection of the test solution containing myristic acid (C14:0), palmitic acid (C16:0), and stearic acid (C18:0) using the Symmetry Shield RP8 column (100 × 3.0 mm, 3.5 µm), Synergi Max-RP C_12_ column (100 × 4.6 mm, 4 µm), Hypersil Gold C_18_ column (150 × 2.1 mm, 3.0 µm), and the Kinetex Evo C_18_ column (150 × 4.6 mm, 2.6 µm).

On the other hand, regarding the selection of the stationary phase as the core of the chromatographic separation, a screening with four RP columns (different chemistry and different dimensions) was carried out. Using a mix of the homologous series composed of myristic acid, palmitic acid, and stearic acid and the gradient program of Ilko et al. [21] (see Table S1), a C_8_, a C_12_, and two C_18_ columns were compared. Since the Hypersil Gold C_18_ columns showed the best results in terms of retention and peak shapes (see Figure 3 and Table S3), and at the same time had the lowest level of background current of the CAD, this column was selected for further experiments. Retention times obtained using the other C_18_ core–shell column were notably longer (no elution of stearic acid within the gradient program) and, interestingly, greatest with the C_12_ column (see Figure 3). Compared to the other columns, this column has the highest carbon load and a considerably higher specific surface area, both of which are factors that influence retention [24]. With the C_8_ column, the three FAs were separated but showed inferior peak shapes and worse separation performance (see Table S3).

#### 2.2.2. Fixing Some of the High-Risk Factors at Reasonable Levels

After fixing the stationary phase and the type of organic solvent, it was necessary to optimize the factors of subsequent importance/factors also posing high-risk toward baseline separation.

The addition of formic acid to the mobile phase is on the one hand necessary for the peak shape, on the other hand it affects the CAD’s level of background current [29]. The concentration of 0.05% (*v*/*v*) was an acceptable compromise that ensures robust protonation of the FAs and a low level of background current (<1 pA). A decrease to 0.02% (*v*/*v*) was associated with peak tailing and an increase in the symmetry factor to values above 1.20.

In the next step before optimization by RSM, the initial and final ACN percentage of the gradient program should be defined. Since data from the scouting stage were available, these high-risk factors could be efficiently fixed to meet practical requirements. Thus, the FA test mixture was subjected to a gradient from 65% (*v*/*v*) to 85% (*v*/*v*) ACN in 15 min at 20 °C and 0.7 mL/min. Under these conditions, a baseline separation between all analytes was achieved, but the elution window was relatively wide and a large expenditure of time that preceded the first-eluting peak, i.e., that followed the last-eluting compound, was evident (see Figure S1a).

To remove empty space before the elution of the least retained FA, the initial and final percentages of ACN in the mobile phase required slight adaptation. It was calculated that at the retention time of the linoleic acid (4.94 min) and the stearic acid (13.23 min), the mobile phase contained approximately 69.8% (*v*/*v*) and 80.8% (*v*/*v*) of ACN, respectively. This calculation was performed using the Equation (1):

(1) $$\varphi_{e}=\varphi_{i}+\frac{\varphi_{f}-\varphi_{i}}{t_{grad}}\times (t_{r}-t_{D})$$

where $\varphi_{e}$, $\varphi_{i}$, and $\varphi_{f}$ are the content of organic solvent at the elution, the beginning of gradient, and the end of gradient, respectively. In the same equation, $t_{grad}$, $t_{r}$, and $t_{D}$ refer to the gradient time, the retention time of the least (or most) retained analyte, and the dwell time (≈ 1.37 min at 700 µL/min), respectively. For simplicity, the initial ACN percentage was rounded to 70% (*v*/*v*) while the final ACN percentage was rounded to 80% (*v*/*v*). Consequently, the gradient range on Hypersil Gold C_18_ stationary phase was modified from 65–85% (*v*/*v*) ACN to 70–80% (*v*/*v*) ACN. In the following test run with the modified gradient conditions, acceptable chromatographic behavior was achieved for all the analytes (see Figure S1b).

#### 2.2.3. Fine-Tuning of CMPs via RSM

Once the combination of initial and final percentage of ACN in the mobile phase was set, secondary parameters were optimized to improve the separation. After subjecting model mixture to adopted settings, it was noted that the critical peak pairs were formed by α-linolenic acid and myristic acid (peaks 1 and 2), i.e., oleic acid and petroselinic acid (peaks 6 and 7). Hence, separation criteria, *S*_1–2_ and *S*_6–7_ between the adjacent peaks were proclaimed as the first two CMAs. Separation criterion *S* is recognized a convenient way of measuring the baseline separation in gradient RP−LC. This is achieved by avoiding some of the disadvantages that standard parameters, such as resolution and peak capacity, suffer, according to [30]. *S* criterion is calculated using Equation (2):

(2) $$S=t_{2,start}-t_{1,end}$$

In Equation (2) $t_{2,start}$ represents the beginning of the later peak, while $t_{1,end}$ is the end of the former peak of two consecutive peaks ($t_{2,start}$ > $t_{1,end})$.

To further fulfill the definition of the ATP, a third CMA was taken into consideration. It was related to the retention of the last-eluting peak, and quantified via the retention factor *k*.

When observed all together, the satisfactory separation of all critical pairs and the reasonable retention of the last-eluting peak were identified as the goals of utmost importance and the supreme ATP of the proposed method. To fulfill these pre-defined CMAs, we varied the flow rate (*x*_1_), the gradient time (*x*_2_), and the column temperature (*x*_3_), as variables that, besides the above-considered (categorical and numerical) factors, highly impact the RP−LC behavior [24,31,32]. These factors were thus declared as relevant CMPs and were subjected to RSM experiments. In order to adequately describe the experimental space, registered CMPs were simultaneously varied according to a face-centered Central Composite Design (CCD) requiring 18 runs in total (see Table 1). Broader ranges of the listed factors were used to examine as wide a space as possible. Clearly, these conditions constituted a trade-off between baseline separation and reduced analysis run-time.

Using Equation (2) the first two CMAs, *S*_1–2_ and *S*_6–7_, were calculated and direct modeling of *S* criteria were attempted. However, this resulted in models with poor predictive performance. One possible reason for the inadequacy of direct modeling could lie in the fact that baseline separation depends on multiple factors (e.g., the size and shape of neighboring peaks). Different assessment of *S* criterion, in this regard, likely comes to the fore in the case of small time differences. The impact of CMPs on the mentioned CMAs was therefore modeled indirectly, which has the advantage that it does not suffer from faulty estimation of baseline separation [33]. Indirect modeling means that mathematical models were developed for the retention times on the chromatogram that correspond to the end of the first peak and the beginning of the second peak. After developing the mathematical models for the corresponding retention times, they were used to calculate separation criteria *S*. The baseline separation was achieved when *S* criterion was greater than zero. On the other hand, the third CMA, *k* of the last-eluting peak, was modeled directly (Table 1). Since it carried information about the analysis runtime, the goal was to minimize its value.

In order to develop desired RSM models, empirical mathematical functions $y=f(x_{1},x_{2},x_{3})$ were fitted to experimentally acquired data [7,34]. Mathematical models are obtained by applying multiple regression and the least squares method, resulting in polynomial equations. The general form of the polynomial expression is given by Equation (3):

(3) $$y=b_{0}+\sum_{i=1}^{3}b_{i}x_{i}+\sum_{i=1}^{3}\sum_{j=1}^{3}b_{ij}x_{i}x_{j}+\sum_{i=1}^{3}b_{ii}x_{i}^{2}$$

In the present function, *y* is the modeled response value (CMA), *x*_i_ indicates independent variable values (*x*_1_, *x*_2_, *x*_3_ represent CMPs as in Table 1), and *b*-s are model coefficients which indicate the magnitude and the trend of respective equation term’s influence, with the exception of *b*_0_, which is the intercept. Thus, *b*_i_, *b*_ij_, and *b*_ii_ indicate single-factor, two-factor interaction (where i ≠ j), and second-order value of factor effects [34].

The model development process was governed by the analysis of variance, which compares level-dependent and random error variances and quantifies model quality by the adjusted coefficient of determination (adj. R^2^) [34,35]. A high value of adj. R^2^ implies concurrence of predicted and measured response values in form of explained variance ratio taking into account degrees of freedom [36] and can be tuned by excluding uninformative model terms. Model validation is often performed with the statistical assessment of lack of fit (*p* > 0.05), a numerical estimation similarity of residual and experimental variance [31,35]. Obtained mathematical models with quality assessment parameters are presented in Table 2. The mathematical transformations of results were applied in line with the Box-Cox plot results. 

### 2.3. Computation of the MODR via Monte Carlo Simulations

The combination of CMP values providing an optimal chromatogram could be estimated simply by overlapping the collected response surfaces from generated RSM models, but this method does not follow the risk management approach [35]. Given that the ICH Q8 guideline stated that the design space (DS) is a multidimensional combination of input variable values and parameters that ensure method quality, it is essential to apply probability-based tools for desired response estimation [23]. As postulated in the ICH Q9 and Q14 guidelines, the definition of DS within analytical method development should be accompanied with quality risk analysis as well as setting up of an appropriate risk control strategy [3,26]. In that respect, the zone of theoretical robustness or an MODR needs to be further defined using statistical tools based on the understanding of the CMAs’ measurement uncertainty and the quantification of the risk of reaching the predefined ATP [3,24,33,35]. The initial step is the discretization of experimental space and creation of uniformly distributed grid points for gradient time [8:0.35:15], flow rate [0.5:0.01:0.7], and column temperature [30:1:40]. Thus, a total number of combinations of CMPs was 21 × 21 × 11 = 4851. To achieve the assurance of quality in terms of meeting predefined acceptance criteria set by the ATP with desired probability, a Monte Carlo (MC) simulation was performed. It included 5000 iterations to propagate the error in model coefficients’ calculation when the error distribution equal to the calculated standard error was added to the estimated model coefficients. In this way response distribution was obtained for each operating condition corresponding to the created 4851 grid points. The criteria for satisfactory CMAs’ values were set as follows: *S*_1–2_ > 0, *S*_6–7_ > 0, and *k*_8_ < 18.8, and MODR was computed for the probability of 90% to meet defined criteria.

**Figure 4:**
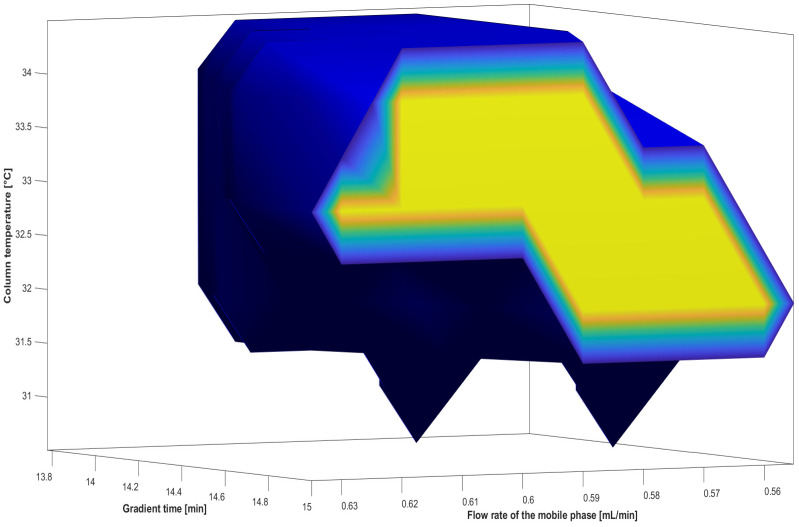
Graphical representation of MODR obtained using Monte Carlo simulations.

**Figure 5:**
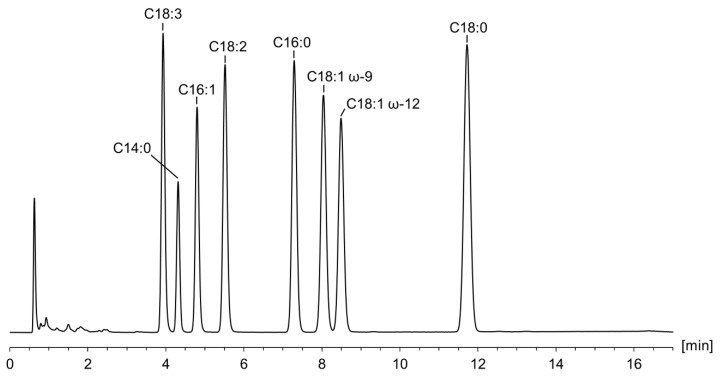
Experimentally obtained chromatogram at selected working values of CMPs (0.575 mL/min flow rate, 14.2 min gradient time, and 33 °C column temperature) with optimized CAD settings (25 °C evaporation temperature, 3.6 s filter constant, and 5 Hz data collection rate).

From the acquired MODR graphical presentation (Figure 4), the margins of the safe zone of theoretical method robustness may be extracted, pointing out to the limits to which the reaching of the ATP would not be compromised. The whole MODR central figure is considered as a “safe zone” from which the working point can be selected randomly. However, it is recommendable to select the working point from the central part (illustrated in Figure 4 in yellow) since the blue colored part represents the edges at risk of falling out of the robust region. Setting up these boundaries is needed for defining a proper method control strategy as required by the ICH Q9 guideline [26]. The working point (0.575 mL/min flow rate, 14.2 min gradient time, and 33 °C column temperature) was further selected from the center of the MODR in order to additionally contribute to method robustness. Figure 5 shows a chromatogram obtained experimentally at the selected working point.

### 2.4. Validation and Application

#### 2.4.1. Validation

The final chromatographic method (see Section 2.3) was validated with regard to guideline ICH Q2 (R1) [37]. In the context of this, specificity, linearity and range, accuracy, repeatability, and limit of quantitation (LOQ) were investigated. When using the AQbD approach, an experimental evaluation of robustness is not mandatory as long as one works within the MODR framework (see Section 2.3) [7,10].

**Figure 6:**
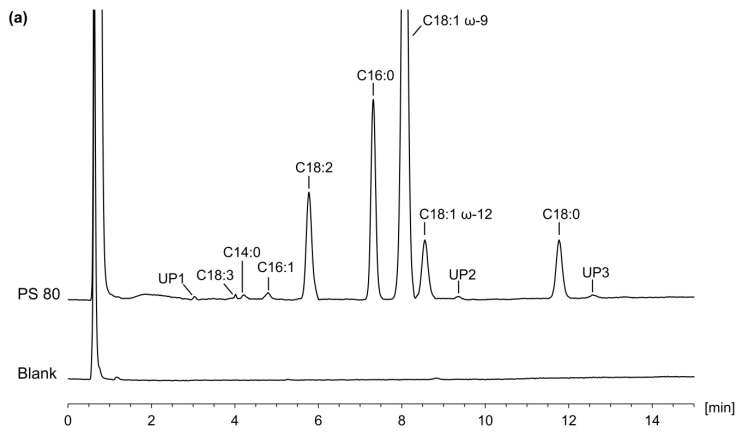

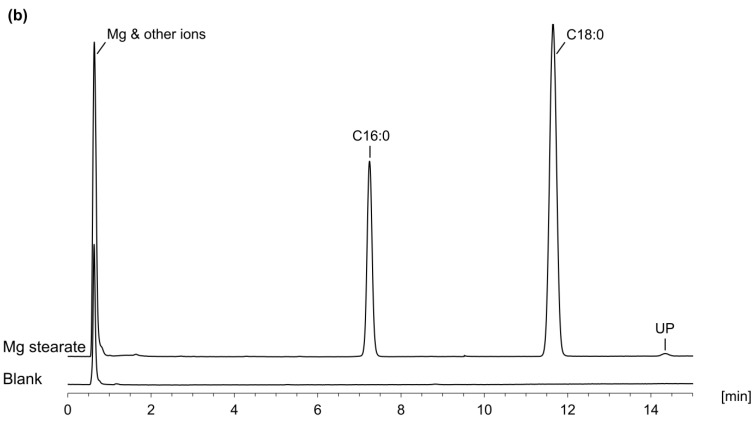
Chromatogram overlay of the application examples with the corresponding blank extraction sample using the final LC-CAD method (see Section 3.3). (**a**) Analysis of polysorbate (PS) 80 with three additional unknown peaks (UP) in addition to the specified non-volatile fatty acids (FA). (**b**) Analysis of magnesium stearate with another unknown peak in addition to two the main FAs palmitic (C16:0) and stearic acid (C18:0).

Specificity was demonstrated by analyzing a mixture of the eight FAs of interest, namely α-linolenic acid (C18:3 ω-3), myristic acid (C14:0), palmitoleic acid (C16:1 ω-7), linoleic acid (C18:2 ω-6), palmitic acid (C16:0), oleic acid (C18:1 ω-9), petroselinic acid (C18:1 ω-12), and stearic acid (C18:0). As shown in Figure 5, all analytes were baseline separated, achieving values of 0.11 or 0.06 for the *S* criterion of the two critical peak pairs α-linolenic acid and myristic acid, and oleic acid and petroselinic acid, respectively. In addition, blank extraction samples were analyzed and checked for possible co-elutions (see Figure 6).

Linearity and range were also determined. To cover the estimated analyte amount of the samples, concentrations of 1, 25, 50, 75, and 100 µg/mL were injected (n = 3) to obtain calibration curves over a range of two orders of magnitude. Different PFVs were tested, but analogous to Schilling et al. [22], the best coefficients of determination (R^2^) were obtained after double logarithmic transformation of concentration and peak area (Table 3).

Accuracy was demonstrated by calculating recoveries at concentration levels of 1, 50, and 100 µg/mL for each fatty acid considered (n = 3) using the log–log calibration curves and ranged from 90% to 108%. Relative standard deviations (RSD) for triplicate injections of FA standards (1, 50, and 100 µg/mL) were used to evaluate repeatability. RSD values between 0.05% and 0.91% indicated satisfactory precision of the method.

Based on the signal-to-noise (S/N) ratios obtained at the calibration level of 1 µg/mL, dilutions of the FAs were prepared to obtain solutions with a S/N of 10:1. The LOQs for each analyte were less than 2 ng on the column (see Table 3). This improvement compared to the UPLC method [22] is probably due to the lower flow rate (0.575 vs. 1.5 mL/min) and thus lower level of background noise. As expected, the highest value was determined for myristic acid (1.85 ng/column) as the shortest-chain FA examined. This reduced detector response is also evident from the high value of the correction factor compared to the other FAs (with respect to oleic acid).

#### 2.4.2. Application Examples

To investigate the performance of the HPLC-CAD method developed here for the analysis of non-volatile FAs using the AQbD approach, we selected magnesium stearate as another application example in addition to PS 80.

In the chromatogram overlay of the PS 80 sample with the blank extraction, no interfering peaks with the reagents or other PS 80 components were visible (see Figure 6a), which was considered as evidence of specificity (see above Section 2.4.1). In addition to the peaks of the indicated eight FAs, three unknown peaks were detectable at 3.0 min, 9.3 min, and 12.6 min with an S/N of just above 10. If unknown signals appear in future samples, especially if their signals increase, an MS analysis for structure elucidation can be performed with the proposed method without adjustments. These three unknown peaks had a combined percent area of only 0.62%. As shown in Table 4, the tested batch of PS 80 complies with Ph. Eur. FA compositional requirements [15]. As already discussed by Ilko et al. [21], the pharmacopoeial method does not distinguish between the two C18:1 isomers oleic acid (ω-9) and petroselinic acid (ω-12). However, since the percentage content of this additional unknown FA is higher at 6.35% compared to the PS 80 batches examined at that time (<3%), it could be useful to introduce a specification for quality control in the future.

Due to poor solubility in water and pure organic solvents, magnesium stearate was dissolved directly in 100.0 mL of a mixture corresponding to the initial gradient conditions and treated for 10 min in an ultrasound bath before analysis. Specificity was similarly ensured by comparison with the chromatogram of the blank sample (see Figure 6b). In the chromatogram of the test solution, besides a large injection peak and the peaks of palmitic and stearic acid, only one additional unknown peak at the end of the gradient was detected. The high intensity of the injection peak is mainly due to the magnesium ions, which are not retained on the C_18_ column used and therefore do not interfere with the determination of the FAs. A quantitative, chromatographic determination of magnesium ions as an alternative to titration has already been demonstrated for magnesium stearate with the nano quantity analyte detector [38] as another type of aerosol−based detector and would theoretically also be possible using the CAD with, e.g., a suitable mixed-mode column [39]. The investigated Mg stearate sample fulfills the requirements of the Ph. Eur. [18] (see Table 4) and the LC-CAD method should also be a simplification for the other monographed salts of stearic acid (i.e., Na, Ca, Zn, and Al). The advantages are not only time savings (no derivatization and shorter analysis time), but also a less error-prone procedure that does not require toxic boron trifluoride as a catalyst.

## 3. Materials and Methods

### 3.1. Chemicals and Reagents

Acetonitrile (ACN) for HPLC (gradient grade, ≥99.9% GC), methanol (MeOH) for HPLC (gradient grade, ≥99.9% GC), *tert*.-butyl methyl ether for HPLC (plus grade, ≥99.9% GC), formic acid MS-grade (≥99.9%), palmitic acid (≥99% GC), stearic acid (≥98.5% GC), oleic acid (≥99% GC), petroselinic acid (≥95% GC), linoleic acid (≥99% GC), α-linolenic acid (≥99% GC), palmitoleic acid (≥98.5% GC), PS 80 (meets specifications of the Ph. Eur. 10.8), magnesium stearate (meets specifications of the Ph. Eur. 10.8), and magnesium acetate × 4 H_2_O (≥99%) were purchased from Sigma Aldrich (Steinheim, Germany). Myristic acid (≥99% GC) and potassium hydroxide for analysis was bought from VWR (Darmstadt, Germany). Ultra-pure water was freshly prepared by a water purification system from Merck Millipore^®^ (Darmstadt, Germany).

### 3.2. Preparation of Solutions and Samples

#### 3.2.1. Standard Solutions for Method Development

For the preparation of the individual stock solutions, 10.0 mg of the respective FA was exactly weighed and dissolved in 10.0 mL MeOH. For the column screening, a test solution of 0.1 mg/mL each of myristic acid, palmitic acid, and stearic acid in a mixture of water:ACN (25:75, *v*/*v*) was used. Additionally, a mixture containing all FAs was prepared at a concentration of 0.1 mg/mL by diluting the stock solutions with the same water/ACN mixture. The obtained solution served as a test solution for scouting and fine-tuning experiments (see Section 2.2.2 and Section 2.2.3). All stock solutions were stored at −20 °C and used throughout the study. The working solutions were prepared daily. In the validation experiments (see Section 2.4.1) and the application examples, a solvent mixture containing water:ACN (30:70, *v*/*v*) with 0.05% (*v*/*v*) formic acid was used for all solutions.

#### 3.2.2. Application Examples—FA Composition in PS 80 and in Magnesium Stearate

Analogous to Ilko et al. [21] a modified saponification and extraction process was employed. In 10.0 mL of a 10% methanolic (*v*/*v*) 1 M KOH solution, 15 mg of the PS 80 (n = 3) was incubated at 40 °C for 6 h. Subsequently, 250 µL of this solution was mixed with 50 µL formic acid in a glass centrifuge tube (VWR, Darmstadt, Germany) to obtain an acidic pH value. After addition of 500 µL *tert*.-butyl methyl ether and vortexing, an incubation period of 5 min was applied before centrifuging the tubes at 2700× *g* for 5 min (EBA 20 centrifuge, Hettich, Tuttlingen, Germany). Finally, the collected organic phase was evaporated under a stream of nitrogen and the residue was dissolved in 1000 µL of the solvent mixture. A blank sample without PS 80 was prepared according to the procedure described above.

A 0.1 mg/mL test solution was prepared by dissolving 10.0 mg of magnesium stearate in 100.0 mL of the solvent mixture. A reference solution of magnesium acetate × 4 H_2_O (0.05 mg/mL) in water was also analyzed.

### 3.3. HPLC-CAD Conditions and Equipment

All experiments were performed on a Vanquish^TM^ Flex modular chromatographic system (Thermo Fisher Scientific, Germering, Germany) consisting of a dual pump F (two independent ternary solvent blending flow streams in one housing) with an online vacuum degasser, a thermostatted split sampler, a thermostatted column compartment with an active pre-heater, and a diode array detector in-line with a Vanquish^TM^ Horizon CAD. The CAD was supplied with nitrogen gas from a 1010 Corona Nitrogen generator (Peak Scientific Instruments, Inchinnan, UK) connected to the in-house compressed air system. The instrument was controlled, and runs were processed using the Chromeleon^TM^ 7.3 Chromatography Data System (Thermo Fisher Scientific).

Four different columns were tested: Waters SymmetryShield RP8 (C_8_; 100 × 3.0 mm, 3.5 µm), Phenomenex Synergi Max-RP (C_12_; 100 × 4.6 mm, 4.0 µm), Phenomenex Kinetex Evo C_18_ (150 × 4.6 mm, 2.6 µm), and Thermo Fisher Hypersil Gold C_18_ (150 × 2.1 mm, 3.0 µm). For column testing, the gradient program of Ilko et al. [21] (see Table S1) was used. Mobile phase consisted of water and ACN, each containing 0.05% (*v*/*v*) formic acid. The flow rate was 0.6 mL/min and the column temperature was set at 30 °C. Test solutions were injected in duplicate.

The face-centered CCD was applied in this study, as an RSM design, in order to obtain mathematical equations, their coefficients, and related standard errors [30]. Experimental factors and their levels varied in CCD are presented in Table S2, while the plan of experiments obtained according to CCD is displayed in Table 1 (see Section 2.2.3). Components of the mobile phase were degassed in an ultrasonic bath for 15 min prior to use. After each change of LC conditions, the system was equilibrated for at least 10 column volumes, followed by a blank sample (water:ACN = 25:75, *v*/*v*).

In order to achieve the best sensitivity, the final tuning of the CAD operating parameters was conducted. The CAD evaporation temperature, filter constant, and data collection rate were varied within following ranges: 20–40 °C, 1–10 s, and 1–10 Hz, respectively.

The final chromatographic conditions consisted of water and ACN each with 0.05% (*v*/*v*) formic acid as mobile phase components and the Hypersil Gold C_18_ column as stationary phase. The column temperature was set to 33 °C and the injection volume to 10 µL at a flow rate of 0.575 mL/min. The percentage of the organic modifier was linearly increased starting from 70% (*v*/*v*) to 80% (*v*/*v*) within 14.2 min. This was followed by a re-equilibration step of 2.8 min. The CAD evaporation temperature was set to 25 °C, the filter constant to 3.6 s, and the data collection rate to 5 Hz. The PFV was set to 1.0 and a log–log transformation was used during validation experiments.

### 3.4. Tools Used for Generating RSM and MODR

The sequence of the required experimental runs (Table 1) was obtained by the Design Expert 7.0.0 software (Stat-Ease Inc., Minneapolis, MN, USA); it was also used for fitting mathematical models to the collected results and model quality assessment. In order to determine the zone of theoretical robustness based on developed CCD models, their coefficients, and standard errors MATLAB^®^ R2018b (MathWorks, Natick, MA, USA) software was used. In particular, MATLAB served for indirect modeling of CMAs, grid point discretization, running MC simulations (statistical tool that takes into account the probability of meeting defined quality criteria) and, finally, generating an MODR graphical presentation.

## 4. Conclusions

Using a risk-based systematic AQbD approach, a robust and trustworthy HPLC-CAD method was developed for the analysis of eight FAs. The performance of the method was ensured within the defined MODR, allowing a long-lasting analytical method lifecycle where changes in chromatographic conditions within the MODR do not require regulatory notification and revalidation. The applicability of the method for the analysis of non-volatile FAs in PS 80 and magnesium stearate was demonstrated. Its main limitation is the applicability in the analysis of only non-volatile FAs due to the basic operating principles of CADs. However, the main advantages of the proposed HPLC-CAD method are its less error-prone and time-saving nature compared to the pharmacopoeial GC method, and the higher sensitivity compared to the already existing HPLC-CAD method. The future perspective is the development of uniform sample preparation procedure for FA analysis. The powerful AQbD approach could be used in the optimization of potentially critical process parameters such as the saponification of the polysorbate and the subsequent extraction of the FAs.

## Figures and Tables

**Table 1:** Plan of experiments according to face-centered CCD and acquired values of critical method attributes (CMAs).

Critical Method Parameters			Critical Method Attributes				
Flow Rate $\left(x_{1}\right)$ [mL/min]	Gradient Time $\left(x_{2}\right)$ [min]	Column Temperature $\left(x_{3}\right)$ [°C]	$t_{(\alpha -linolenic\;acid)end}$	$t_{(myristic\;acid)start}$	$t_{(oleic\;acid)end}$	$t_{(petroselinic\;acid)start}$	*k*_(stearic acid)_
0.50	8.0	30.00	4.84	4.94	8.99	9.01	15.80
0.70	8.0	30.00	3.53	3.6	6.85	6.85	16.99
0.50	15.0	30.00	4.95	5.07	9.98	10.02	18.78
0.70	15.0	30.00	3.61	3.67	7.47	7.49	19.86
0.50	8.0	40.00	4.31	4.31	7.82	7.82	13.73
0.70	8.0	40.00	3.12	3.12	5.86	5.86	14.64
0.50	15.0	40.00	4.37	4.37	8.47	8.47	15.86
0.70	15.0	40.00	3.15	3.15	6.27	6.27	16.59
0.50	11.5	35.00	4.56	4.61	8.83	8.84	16.41
0.70	11.5	35.00	3.33	3.38	6.66	6.66	17.32
0.60	8.0	35.00	3.84	3.87	7.21	7.21	15.45
0.60	15.0	35.00	3.9	3.95	7.85	7.87	17.88
0.60	11.5	30.00	4.11	4.19	8.22	8.23	18.44
0.60	11.5	40.00	3.62	3.62	7.00	7.00	15.48
0.60	11.5	35.00	3.89	3.93	7.63	7.64	16.65
0.60	11.5	35.00	3.86	3.9	7.57	7.58	16.71
0.60	11.5	35.00	3.85	3.89	7.55	7.57	17.26
0.60	11.5	35.00	3.85	3.91	7.57	7.6	16.87

**Table 2:** Generated DoE models which refer to coded factor values are present in tabular form, along with model quality parameters.

	${1/t}_{(\alpha -linolenic\;acid)end}$	$1/t_{(myristic\;acid)start}$	${log(t}_{(oleic\;acid)end})$	$log(t_{\left(petroselinic\;acid\right)start})$	*k*_8_
*b*_0_	+0.26	+0.26	+0.88	+0.88	+16.89
*b*_1_	+0.041	+0.041	−0.062	−0.062	+0.48
*b*_2_	−2.111 × 10^−3^	−2.196 × 10^−3^	+0.018	+0.019	+1.24
*b*_3_	+0.016	+0.019	−0.035	−0.035	−1.36
*b*_12_	/	/	−1.631 × 10^−3^	−1.582 × 10^−3^	/
*b*_13_	+3.182 × 10^−3^	+3.317 × 10^−3^	−1.506 × 10^−3^	−1.313 × 10^−3^	/
*b*_23_	/	/	−2.369 × 10^−3^	−2.611 × 10^−3^	−0.22
*b*_11_	/	/	+4.776 × 10^−3^	+4.493 × 10^−3^	/
*b*_22_	/	/	−3.539 × 10^−3^	−3.515 × 10^−3^	−0.33
*b*_33_	/	/	/	/	/
R^2^	0.9993	0.9993	0.9997	0.9998	0.9902
Adj. R^2^	0.9991	0.9991	0.9995	0.9996	0.9861
Pred. R^2^	0.9985	0.9987	0.9991	0.9992	0.9805
Lack of fit; *p* value	0.7790	0.6445	0.9730	0.9650	0.9718

**Table 3:** Coefficient of determination (R^2^) of the calibration curve (after log–log transformation), correction factor, and LOQ of the investigated FAs.

	R^2^	Slope	y-Intercept	Correction Factor	LOQ (ng/Column)
α-linolenic acid	0.9873	0.9350	0.0935	1.01	0.38
Myristic acid	0.9995	1.1522	0.0219	1.25	1.85
Palmitoleic acid	0.9955	1.0025	0.0529	1.09	0.91
Linoleic acid	0.9936	0.9419	0.0937	1.02	0.62
Palmitic acid	0.9934	0.9914	0.0921	1.07	0.77
Oleic acid	0.9951	0.9231	0.1128	1.00	0.64
Petroselinic acid	0.9952	0.9343	0.1089	1.01	0.82
Stearic acid	0.9899	0.9540	0.1517	1.03	0.77

**Table 4:** Results of the LC-CAD analysis of a PS 80 and a magnesium stearate sample.

PS 80			Mg Stearate		
Ph. Eur. Monograph		LC-CAD	Ph. Eur. Monograph		LC-CAD
Myristic acid	≤5.0%	0.21%	Stearic acid Palmitic acid Unknown peaks Sum of stearic and palmitic acid	≥40.0% n.s. ^a^ n.s. ^a^ ≥90.0%	69.3% 30.2% 0.5% 99.5%
Palmitic acid	≤16.0%	15.6%			
Palmitoleic acid	≤8.0%	0.47%			
Stearic acid	≤6.0%	5.8%			
Oleic acid	≥58.0%	62.1%			
Petroselinic acid	n.s. ^a^	6.35%			
Linoleic acid	≤18.0%	8.72%			
Linolenic acid	≤4.0%	0.13%			
Unknown peaks	n.s. ^a^	0.62%			

^a^ not specified.

## Data Availability

The data presented in this study are available in the article “Analytical Quality by Design: Achieving Robustness of an LC-CAD Method for the Analysis of Non-Volatile Fatty-Acids” and the corresponding Supplementary Material.

## Supplementary Materials

The following supporting information can be downloaded at https://www.mdpi.com/article/10.3390/ph16040478/s1, Figure S1: Chromatograms of the test solution applying different scouting gradients; Table S1: Gradient program for column screening; Table S2: Investigated levels of the CMPs; Table S3: Peak characteristics of the column screening.

## Abbreviations

ACN	Acetonitrile
ATP	Analytical Target Profile
AQbD	Analytical Quality by Design
CAD	Charged Aerosol Detector
CCD	Central Composite Design
CMA	Critical Method Attribute
CMP	Critical Method Parameter
DoE	Design of Experiments
DS	Design Space
FA	Fatty Acid
*k*	Retention factor
LC	Liquid Chromatography
LOQ	Limit of Quantitation
MC simulation	Monte Carlo simulation
MeOH	Methanol
MODR	Method Operable Design Region
PFV	Power Function Value
PS	Polysorbate
Ph. Eur.	European Pharmacopoeia
QbD	Quality by Design
R^2^	Coefficient of Determination
RP	Reversed Phase
RSD	Relative Standard Deviation
RSM	Response Surface Methodology
S criterion	Separation criterion
S/N	Signal-to-Noise Ratio
