# Supplementary material for: Analytical Quality by Design: Achieving Robustness of an LC-CAD Method for the Analysis of Non-Volatile Fatty Acids
Source: Pharmaceuticals (Basel). 2023 Mar 23;16(4):478. doi: 10.3390/ph16040478 (PMC10145151; doi:10.3390/ph16040478)
Supplement: Supplementary file 1 [file pharmaceuticals-16-00478-s001.zip › pharmaceuticals-2228025-supplementary.pdf]

Supporting information

Analytical Quality by Design: Achieving Robustness of an LC–CAD Method for the Analysis of Non-Volatile Fatty Acids

Rasmus Walther [1,†], Jovana Krmar [2,†], Adrian Leistner [1], Bojana Svrkota [2], Biljana Otašević [2], Andjelija Malenović [2], Ulrike Holzgrabe [1,*] and Ana Protić [2,*]

[1] Institute for Pharmacy and Food Chemistry, University of Würzburg, Am Hubland, 97074 Würzburg, Germany
[2] Department of Drug Analysis, Faculty of Pharmacy, University of Belgrade, Vojvode Stepe 450, 11 221 Belgrade, Serbia
* Correspondence: ulrike.holzgrabe@uni-wuerzburg.de (U.H.); ana.protic@pharmacy.bg.ac.rs (A.P.); Tel.: +49-931-31-85461 (U.H.); +381-1-11-395-1334 (A.P.)
† These authors contributed equally to this work.

Table S1. Gradient program from Ilko et al. [21] for the separation of fatty acids using water and ACN each with 0.05 % (v/v) formic acid as mobile phases A and B, respectively, at a flow rate of 0.6 mL/min and a column temperature of 30 °C

Time [min]	%-A (v/v)	%-B (v/v)
0 – 5	25	75
5 – 15	25 – 15	75 – 85
15 – 18	25	75

Table S2. Investigated levels of the critical method parameters (CMP) for the method optimization by means of design of experiments using a 3 x 3 face-centred central composite design

CMP	Low level (-1)	Nominal level (0)	High level (+1)
Flow rate, X_1 [mL/min]	0.5	0.6	0.7
Gradient time, X_2 [min]	8.0	11.5	15.0
Column temperature, X_3 [°C]	30.0	35.0	40.0

Table S3. Peak characteristics (retention time (RT), capacity factor (k), symmetry factor (A_s), width at half height ($w_{0.05}$), separation factor (α) and resolution (R_s)) of the column screening experiments

Column	Peak	Myristic acid	Palmitic acid	Stearic acid	Background noise
Symmetry Shield RP8 (100 x 3.0 mm, 3.5 µm)	RT	3.305 min	5.080 min	8.083 min	1.3 pA
	k	4.92	8.10	13.49	
	A_s	1.32	1.14	1.18	
	$w_{0.05}$	0.097 min	0.124 min	0.172 min	
	α	1.65	1.66	n.a. [a]	
	R_s	9.44	12.06	n.a. [a]	
Synergi Max-RP C_{12} (100 x 4.6 mm, 4.0 µm)	RT	13.520 min	n.a. [a]	n.a. [a]	2.5 pA
	k	10.31			
	A_s	1.03			
	$w_{0.05}$	0.222 min			
	α	n.a. [a]			
	R_s	n.a. [a]			
Hypersil Gold C_{18} (150 x 2.1 mm, 3.0 µm)	RT	3.252 min	5.647 min	9.828 min	0.9 pA
	k	4.63	8.77	16.0	
	A_s	1.13	1.01	1.02	
	$w_{0.05}$	0.0690 min	0.121 min	0.180 min	
	α	1.90	1.82	n.a. [a]	
	R_s	14.87	16.39	n.a. [a]	
Kinetex Evo C_{18} (150 x 4.6 mm, 2.6 µm)	RT	9.077 min	14.423 min	n.a. [a]	1.1 pA
	k	4.19	7.25		
	A_s	1.07	1.01		
	$w_{0.05}$	0.127 min	0.144		
	α	1.72	n.a. [a]		
	R_s	23.28	n.a. [a]		

[a] not applicable

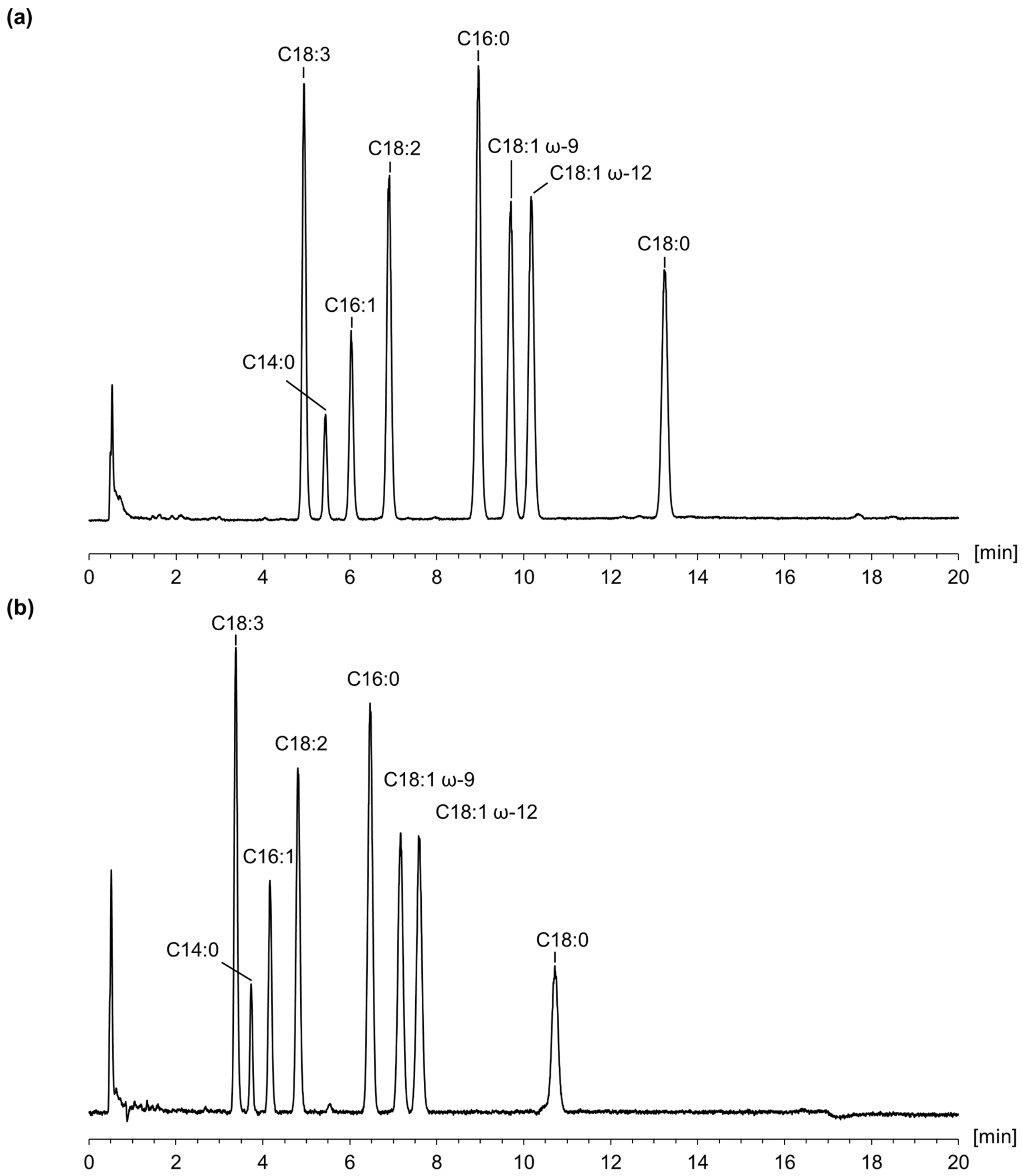

Figure S1. Chromatogram of the test solution containing all FAs (0.1 mg/mL) applying a 15 min gradient program with a flow rate of 0.7 mL/min at a column temperature of 20 °C:
(a) linear increase of the ACN percentage from 65 % (*v/v*) to 85 % (*v/v*)
(b) linear increase of the ACN percentage from 70 % (*v/v*) to 80 % (*v/v*)
